# Aspergillus-derived β-glucan nanoparticles: a dual strategy for *Fusarium Wilt* management and tomato plant growth enhancement

**DOI:** 10.3389/fpls.2025.1611582

**Published:** 2025-07-30

**Authors:** Parthasarathy Ramalingam, Manikandan Appu

**Affiliations:** ^1^ Department of Biochemistry, Indian Institute of Science, Bengaluru, Karnataka, India; ^2^ Department of Biotechnology, RVS Agricultural College, Thanjavur, Tamil Nadu, India; ^3^ Department of Plant Sciences, University of Hyderabad, Hyderabad, Telangana, India

**Keywords:** Aspergillus awamori, β-glucan nanoparticles, tomato wilt, Fusarium oxysporum, detached leaf assay

## Abstract

**Introduction:**

A soilborne Ascomycete, *Fusarium oxysporum f.sp. lycopersicum*, is the causative agent of wilt disease, posing a significant threat to tomato plants and severely impacting global tomato production. Chemical fungicides are the primary strategy for controlling it. Employing fungicides arbitrarily and in huge dosages can pollute the environment and harm field workers and customers.

**Methods:**

To combat tomato wilt, we synthesized β-glucan (isolated from the marine algal associate *Aspergillus awamori*) nanoparticles (β-glu-n) and evaluated their efficacy in promoting plant growth and suppressing *Fusarium oxysporum*.

**Results and discussion:**

The synthesized β-glu-n was confirmed using NMR and IR spectroscopy. The spherical shape with a smooth surface and average size of 35 ± 6.0 nm was observed by TEM. The hydrostatic zeta potential was -38.40 mV, indicating colloidal stability. The crystalline structure of the β-glu-n was confirmed by the XRD spectrum. Furthermore, a significant seed germination and growth profile, including higher shoot and root length and lateral root, was observed in the β-glu-n-treated tomato seeds count than in the mycelial glucan (m-β-glu) and control group under glasshouse conditions. Moreover, novel protein polypeptides were derived from β-glu-n-treated plants, indicating the increased photosynthetic rate. β-glu-n inhibited *Fusarium oxysporum* in a disc diffusion test and reduced wilt symptoms in under detached leaf assay. These results suggest that β-glucan nanoparticles can promote plant growth and prevent tomato wilt disease.

## 1 Introduction

Tomatoes (*Solanum lycopersicum L*.) rank among the most favoured fruits globally and widely consumed vegetables, noted for their nutritional value and economic importance (Quinet et al., 2019; Erika et al., 2020). Their tomato yield was decreasing due to the crops being impacted by the wilt disease, caused by the *Fusarium oxysporum f.sp. lycopersicum* (soil-borne pathogen). This pathogen enters tomato plants through their roots all the growth level stages, producing significant economic losses by causing necrosis and wilting, eventually culminating plant death. Controlling this pathogen remains tough due to its long-term stay in the soil and capacity to infect a large number of host plants (Jangir et al., 2021; El-Aswad et al., 2023; Chakrapani et al., 2023);.

Therefore, it is crucial to investigate an effective and environmentally friendly alternative, offered the considerable interest in biological control agents. These methods control these pathogens through natural bio-resource or bio-based polymers and provides benefits including availability from sustainable agricultural resources, biodegradable properties and ecological safety, which can enhance the plant defense mechanisms. β-glucans are the primary polysaccharides in the cell wall that play a crucial role in inducing systemic resistance (defense mechanism) during pathogen infection (Camilli et al., 2018). It has also been demonstrated that it improves crop protection against pathogens and enhances plant growth development (Riseh et al., 2023; Chavanke et al., 2022).

The emergence of nanotechnology and the advancement of innovative nanomaterials explore potential openings and new applications in agricultural biotechnology (Iavicoli et al., 2017; Shang et al., 2019; Sundararajan et al., 2023). β-glucan nanoparticles have widespread applications in different areas, used as drug delivery systems (Huang et al., 2020), anticancer molecules (Parthasarathy et al., 2021) and biocontrol agents, biofertilizers (Murphy et al., 2022). Their nanoscale dimensions improve bioavailability and surface reactivity, rendering them effective in targeting phytopathogens (Chavanke et al., 2022). Moreover, biopolymer-based nanoparticles, such as glucan and chitosan, have the capacity to improve plant growth development by enhancing nutrient uptake and optimizing photosynthate effectiveness (Photosynthesis) and also inhibiting fungal pathogens specifically, β-glucans nanoparticle can stimulate the activities of key enzyme such as peroxidase and polyphenol oxidases, which are associated with both defence and metabolic efficiency there by potentially enhancing the allocation and utilization of photosynthates rates for plant growth and development (Kaziem et al., 2024; Melo et al., 2020). Recently, Anusuya and Parthasarathy (2025) reported that glucan nanoparticles were synthesised using sodium tripolyphosphate have much attention for their biocompatibility and biocidal properties.

The current study focuses on the preparation of nanoparticles derived from fungal glucan, extracted from the marine algal associated fungi. It determines their effectiveness against wilt disease caused by *Fusarium oxysporum f.sp. lycopersicum* and their capacity to stimulate the growth of tomato plants. The purpose of this research is to modify bio-resources into nanocomposites to provide an effective and eco-friendly method for protection against pathogens and to enhance the growth and development of tomato plants.

## 2 Materials and methods

### 2.1 Pathogen and seeds collection

Tomato wilt pathogen (*Fusarium oxysporum*) was obtained from the Indian Type Culture Collection (ITCC), Indian Agricultural Cultural Research Institute (IARI), New Delhi, India. The pathogen was maintained at 4°C.

Tomato seeds of the PKM-1 variety were received from Tamil Nadu Agricultural University (TNAU), Coimbatore, Tamil Nadu, India.

### 2.2 Collection and transport of *Ulva lactuca*

*Ulva lactuca* (marine green algae) was obtained from the shoreline of Rameswaram, Tamil Nadu, India. Fresh and healthy thalli were carefully handpicked and immediately placed in sterile polythene bags filled with seawater to maintain their physiological condition and prevent dryness. The collected algae samples were carried to the laboratory within 24 hours of collection for endophytic fungi isolation.

### 2.3 Fungal material: isolation and identification

The endophytic fungus was isolated and identified following the method described by earlier reports (Parthasarathy and Sathiyabama, 2014; Parthasarathy et al., 2020). In brief, the fungus was obtained from the marine green algae *Ulva lactuca*. The isolated fungal strain (designated as UL) was cultured on potato dextrose agar (PDA) medium supplemented with sea salts, adjusted to pH 5.1. The cultures were incubated at 30°C for a period of 10-15 days. By the 10th day of incubation, the fungus developed black and white colonies on the surface of the PDA medium (**Supplementary Figure S1**).

Fungal genomic DNA was isolated to enable molecular characterization of the species. The ITS region was targeted for amplification using ITS 1 as the forward primer and ITS 4 as the reverse primer. The resulting amplified ITS product was sequenced and analysed for species identification using the BLAST tool. The analysis revealed a 99% identity with *Aspergillus awamori* (AA). The sequence was subsequently deposited in the NCBI GENBANK database and assigned the accession number MH920241.1 for the fungal species. Additionally, a phylogenetic tree based on the ITS sequence of *A. awamori* was constructed using the neighbor-joining method. The analysis demonstrated a 99.5% similarity to other *A. awamori* species, further confirming the identity of the isolate (**Supplementary Figure S2**).

### 2.4 Extraction of glucan from fungal mycelium

*A. awamori* spores (1x10^5^/mL) were inoculated into potato dextrose broth (PDB) and incubated for 7 days at 27°C. Then, the fungal mycelium was harvested and washed with sterile distilled water. The mycelia were then resuspended in double distilled water (10 mL/g mycelium) and pulverized using a pestle and mortar. The mycelial slurry was filtered through filter paper. The residue was homogenized three times in water, once in a 1:1 combination of chloroform and methanol, and finally in acetone before being dried at 55°C using a rotary vacuum evaporator. The mycelial wall was pulverized into a fine powder using a sterile pestle and mortar before being extracted with 100 mL of 1 M NaOH at 95°C for 2 hours to remove proteins, lipids, and alkali-soluble polysaccharides. The resulting suspension was subsequently allowed to reach room temperature, filtered, and then neutralized with the water. The pellet was rinsed with 95% ethanol (v/v) and freeze-dried. The dried powder was treated with 20% (v/v) HCl (50 ml/g dry matter) overnight at 60°C and stirred. The precipitate was neutralized with distilled water, freeze-dried, and kept at 4°C to prepare nanoparticles (Anusuya and Sathiyabama, 2014; Sathiyabama and Charles, 2015).

### 2.5 Synthesis of glucan nanoparticle from mycelial glucan

1 g of mycelial glucan (m-glu) was dissolved in 100 ml of 2% NaOH (w/v) while maintain controlled magnetic stirring for 3 hours at 90°C. At the end of incubation period, β-glucan nanoparticles (β-glu-n) were precipitated using 1% (v/v) acetic acid. To prepare a stable suspension of 5mg/mL β-glu-n, the solution was added dropwise to a tripolyphosphate (2mg/mL) solution while stirring magnetically at room temperature. The suspension was then stirred for an additional hour at room temperature before being centrifuged at 5000 rpm for 20 minutes. The pellet was washed with MilliQ water to remove debris and stored at 4°C for characterization and bioactivity assessment (Parthasarathy et al., 2021).

### 2.6 NMR analysis of mycelial β-glucan and β-glucan nanoparticles

The m-β-glu and β-glu-n were analyzed using NMR to confirm the β glucan and its nano-reductant. The m-glu and β-glu-n (5mg) were dissolved in DMSO-D6and transferred to an NMR glass tube associated with the NMR JEOL apparatus. The 500 MHz spectrometer was employed to acquire Nodel ECZ500 spectra at a stable temperature of 80°C. The ^1^H NMR spectra were obtained within a scan range of 2000 (0-7 ppm).

### 2.7 IR spectrum analysis of β-glu-n

The prepared m-β-glu and β-glu-n were ground into fine powders and mixed with KBr to form pellets. The pellets underwent analysis with a Thermo-Nicolet 6700 FTIR spectrophotometer, covering the infrared range of 400-4000 cm^-1^.

### 2.8 Ultraviolet spectral analysis of β-glu-n

The SPR (surface plasmon resonance) of β-glu-n was measured using a UV-Vis spectrometer (J Shimadzu MPC3600) at 200-500 nm.

### 2.9 Assessment of size and zeta potential for β-glu-n

The β-glu-n was dissolved in deionized water and subsequently transferred into a glass cuvette, where it was positioned on the ZETA PALS. The temperature remained constant at 25°C throughout the entire process. The sizes of nanoparticles and their distribution range were recorded, as well as the values for the polydispersity index (PDI) were measured.

#### 2.9.1 HR-TEM and SEM analysis of β-glu-n

The morphology and size of β-glu-n were assessed using a scanning electron microscope (FEI ESEM QUANTA 200) and transmission electron microscopy (TEM). Desiccated β-glu-n were positioned on the copper grid of the Titan Themis 300 kV FEI Transmission Electron Microscope (ULTRA 55-GEMINI technology). The size, structure, and micrograph of the nanoparticles were captured.

#### 2.9.2 X-ray diffraction pattern of β-glu-n

The dried β-glu-n were placed on a glass slide, and the sample was analyzed using the Rigaku Smart Lab general-purpose X-ray diffractometer system (utilizing XPERT Pro, PANalytical JDX-8030, and JEOL). The diffraction intensities of the nanoparticles were measured across a 2θ angle range from 10 to 90°.

#### 2.9.3 Invitro assessment of β-glu-n on *F.oxysporum* growth

*In Vitro* assessment of β-glu-n *F. oxysporum* inhibition potential was assessed on PDA plates using the disc diffusion method. β-glu-n and m-glu were dissolved in sterile distilled water (100µg/mL). Five-day-old *F.oxysporum* mycelial plugs (6-mm-dm) were placed on a PDA plate. A sterile disc (5mm-dm) loaded with β-glu-n (50µl) and m-glu (50µl) was placed alongside the *F. oxysporum* culture and incubated at 24°C for 48 hours. The disc with sterile distilled water was employed as a control.

#### 2.9.4 Efficacy of β-glu-n on disease development assessed using detached leaves assay

The effectiveness of β-glu-n in controlling *F. oxysporum*-induced wilt disease in tomato plants was examined using the detached leaves assay (Manikandan and Sathiyabama, 2016). To do this, 25-day-old tomato leaves were carefully plucked off the plants with sterile blades. The leaves were cleaned adequately with running tap water to remove debris, followed by two washes with sterile distilled water *in vitro*. Following sterilization, the leaves were placed in petri dishes and covered with filter paper for 5 minutes to remove excess moisture. Sterilized leaves were treated with 0.1% (w/v) β-glu-n and m-glu (50 µl/leaf) using a sterile acrylic brush and 5 ml of sterile distilled water were treated as a control. The leaves were incubated at 25 ± 2°C for 24 hours. After 24 hours, treated and control leaves were challenged with a spore suspension of *F. oxysporum* (1×10^5^ spores/ml) (100 µl/leaf) applied with a sterile acrylic brush. To assess disease progression, treatment and control leaves were kept at 25 ± 2°C and 100% humidity for 15 days.

#### 2.9.5 The effect of β-glu-n on plant development in a greenhouse environment

Tomato seeds treated with 5ml of 0.1% (w/v) β-glu-n and m-glu were placed in poly plant growing bags (12 x 12 inches) containing soil, and infected with spores of *F. oxysporum* (1×10 spores’ ml^-1^) in a greenhouse environment. Tomato plants grown in soil with solely *F. oxysporum* spores (1×10 spores’ ml^-1^) served as the control group. Growth profiles including shoot length, root length, and leaf count were meticulously recorded in both treated and control plants. Each experiment utilized 50 plants, with three repeats performed.

#### 2.9.6 Implications of β-glu-n on protein profile in tomato leaves

The treated and control tomato leaves were collected on 25^th^ day. Leaves were homogenised in 0.02 M sodium acetate buffer and centrifuged at 10,000 rpm for 20 minutes at 4°C. The protein content in the supernatant was determined using the Bradford method (Bradford, 1976). The samples (30 µg) underwent denaturation for five minutes in a boiling water bath with SDS and sample buffer, followed by separation with marker proteins (Bradford, 1976). The localization of polypeptides on SDS PAGE was achieved through staining with Coomassie Brilliant Blue.

## 3 Results and discussion

Continuous innovation in agriculture is necessary to address present obstacles including food security, and climate change. This expands scope of nanotechnology as a new source for improving existing crop management techniques (Shang et al., 2019; Ahmad et al., 2024). The use of nanomaterials, particularly those based on natural source is becoming more popular due to their high specificity and improved functionality, and typically excellent biocompatibility. Glucan nanoparticles, in particular are valued for their consistent structure and widely applied in fields including drug delivery and the agricultural sector (Kaziem et al., 2022; Chen et al., 2024).

### 3.1 Characterization of β-glucan nanoparticles

This research focused on the synthesis of mycelial glucan nanoparticles via biological method using sodium tripolyphosphate (TPP). A total of 15 g of mycelial β-glucan was extracted from *A. awamori*. Additionally, the synthesis of β-glu-n, specifically mycelial glucan, was achieved by solubilizing in NaOH at ambient conditions. The resulting nanoparticles were formed through electrostatic interaction between the glucan and tripolyphosphate (TPP), with their shape, structural uniformity and stability being influenced by these interactions.

**Figure 1:**
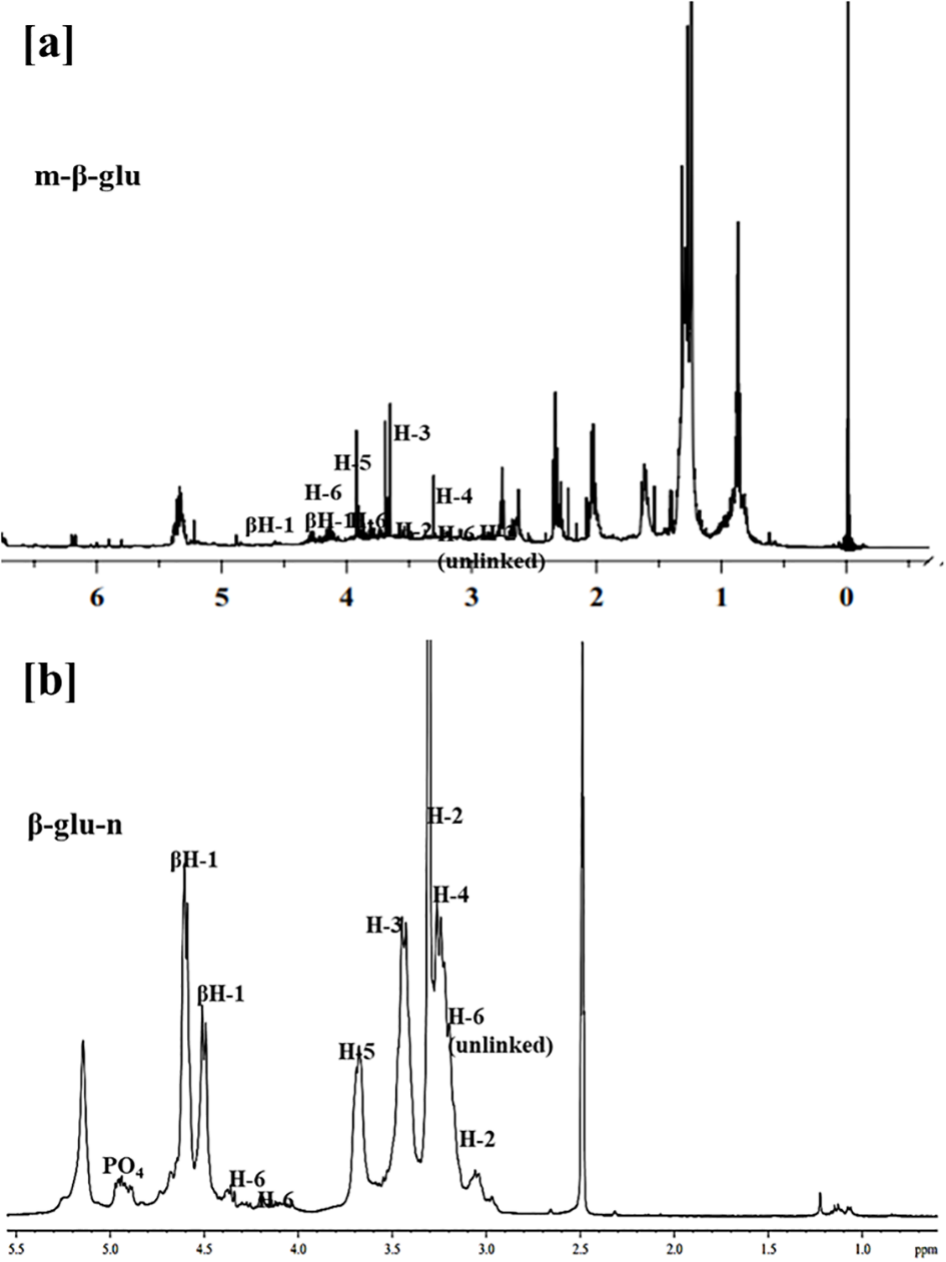
**(a, b)** ^1^H NMR spectrum of **(a)** m-β-glu **(b)** synthesized β-glu-n with tripolyphosphate (TPP).

The typical 1H NMR chemical shifts for carbohydrate ring proton range from 3-6 ppm. The synthesis of β-glu-n was corroborated by 1H NMR signals corresponding to m-β-glu (4.35,4.58) and β-glu-n (4.50,4.68 ppm), which indicated of β-1,6 linkages (**Figures 1a, b**). A signal at 3.39ppm appeared exclusively for nanoparticles and was ascribed to the H-1 phosphate connected to the α-anomer at the reducing end. These 1H signals assignments align with values previously documented values for analogous compounds (Kim et al., 2000; Parthasarathy et al., 2021). 

**Figure 2:**
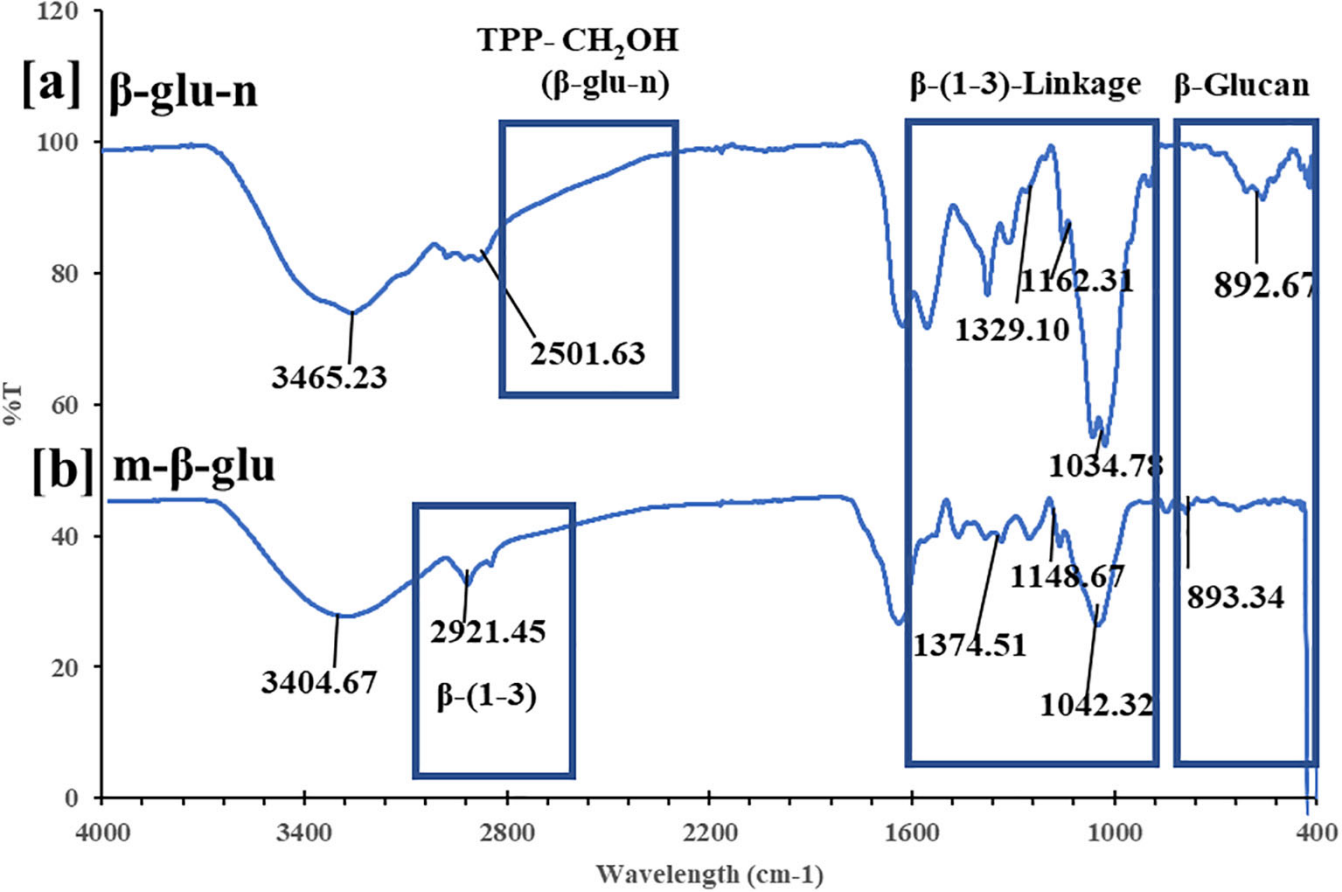
**(a, b)** IR spectrum analysis of **(a)** β-glu-n **(b)** m-β-glu.

The IR spectrum of β-glucan exhibited a characteristic pattern typical of carbohydrate moieties, with several bands observed in the anomeric area. The β-glu-n and m-glu IR spectra displayed a distinctive absorption peak at 892.67 and 893.34 cm^-1^, respectively, indicative of β-glucan (**Figure 2a**). Moreover, m-glu and β-glu-n peaks were observed at 2921.45, 1374.51, 1148.67, 1042.32 cm^-1^, and 1329.10, 1162.31, 1162.31,1034.78 respectively, signifying β-(1-3) linkage. The β-glu-n was changed through the regulated incorporation of TPP, which conferred structural integrity and stability to the nanoparticles. A peak at 2508.63 cm^-1^, resulting from the OH-P=O stretch, can be ascribed to the connection between the phosphoric group of TPP and the CH_2_OH group of the β glu-n (**Figure 2b**) (Limberger-Bayer et al., 2014; Bacha et al., 2017; Mahmoud and Yassein, 2024)

The UV-visible spectrum exhibited a prominent surface plasmon resonance (SPR) peak at 389.45 nm, confirming the synthesis of β-glu-n stabilized at ambient temperature (**Supplementary Figure S3**).

**Figure 3:**
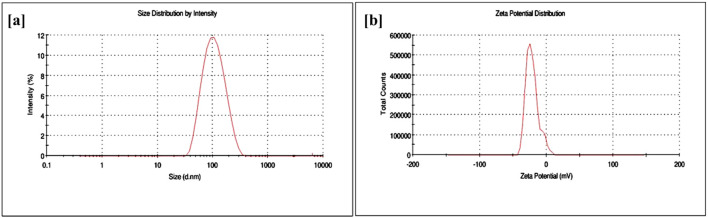
**(a, b)** Particle size **(a)** and zeta potential **(b)** analysis of β-glu-n.

DLS demonstrated the average size of the β-glu-n in the range of 85.3 nm, with a narrow size distribution and strong dispersion (Polydispersity Index of 0.284) (**Figure 3a**). The zeta potential was -38.40 mV, demonstrating colloidal dispersion of the nanoparticles (**Figure 3b**). The detrimental impact could be due to TPP binding, which produces repellent effects on nanostructures (Wu et al., 2005; Khlebtsov and Khlebtsov, 2011). The low index of polydispersity indicates a uniform size range of the nanoparticles, reflecting effective fabrication and stability. This results in particles that are less prone to aggregation and demonstrate consistent behaviour in different conditions (Ashraf et al., 2021).

**Figure 4:**
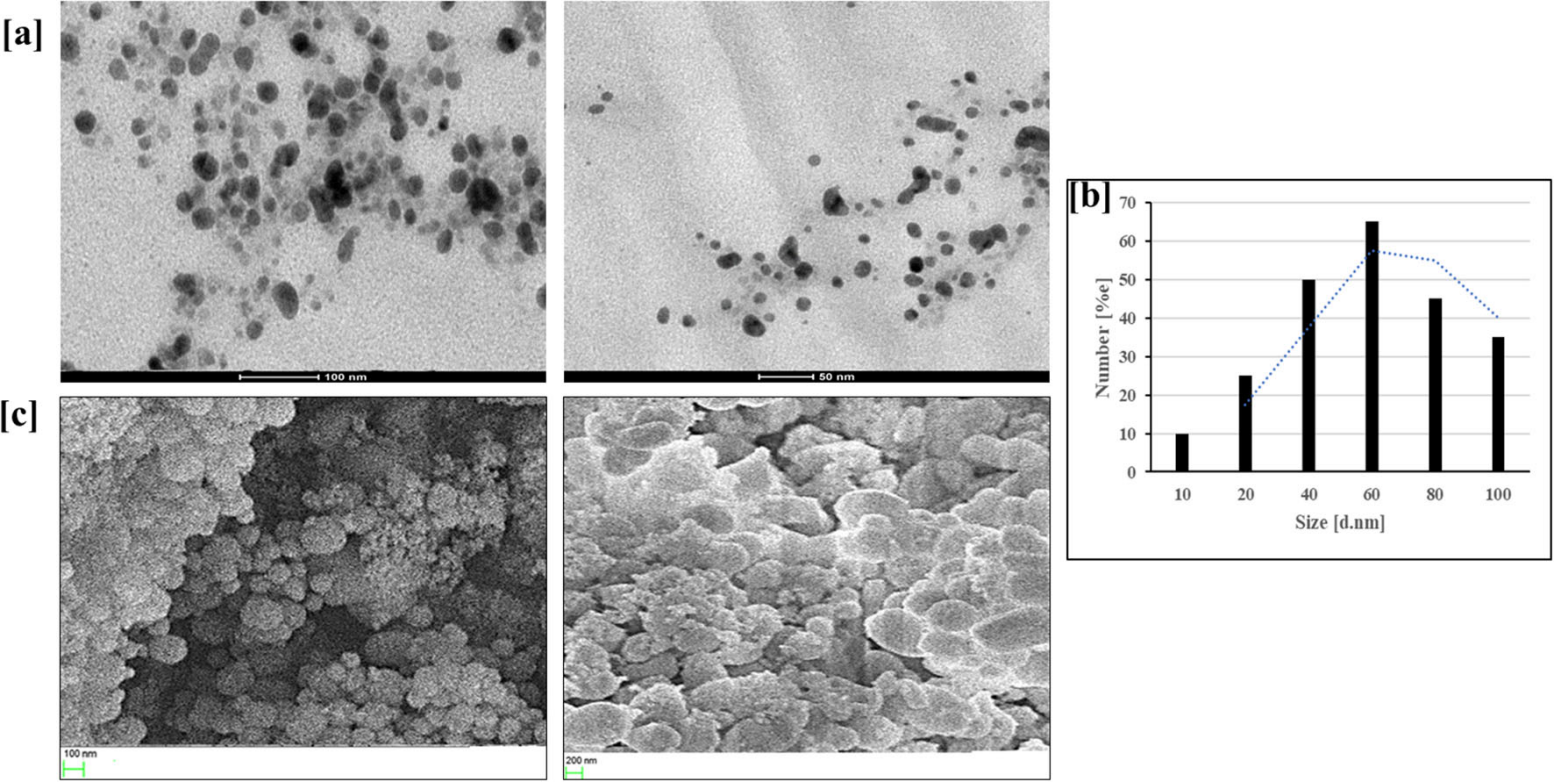
**(a–c)** HR-TEM images of β-glu-n **(a)**, particle size distribution histogram of β-glu-n **(b)**, SEM micrograph of β-glu-n **(c)**.

HR-TEM images showed a uniform arrangement of β-glu-n, with an average size of 35 ± 6.0 nm (**Figures 4a, b**). The β-glu-n exhibited a colloidal nature, which was indicated by a spherical shape and a smooth surface, revealing long-term durability. The SEM images also displayed a round morphology with a smooth surface (**Figure 4c**). Both SEM and TEM demonstrated an even distribution of nanoparticles, primarily attributed to the presence of TPP in the synthesis process (Piani and Papo, 2013). The HR-TEM and DLS size of the nanomaterials exhibited variance due to the different principle and concepts involved. The synthesized glucan nanoparticles underwent modification through the controlled addition of TPP, enhancing their structural integrity and stability.

**Figure 5:**
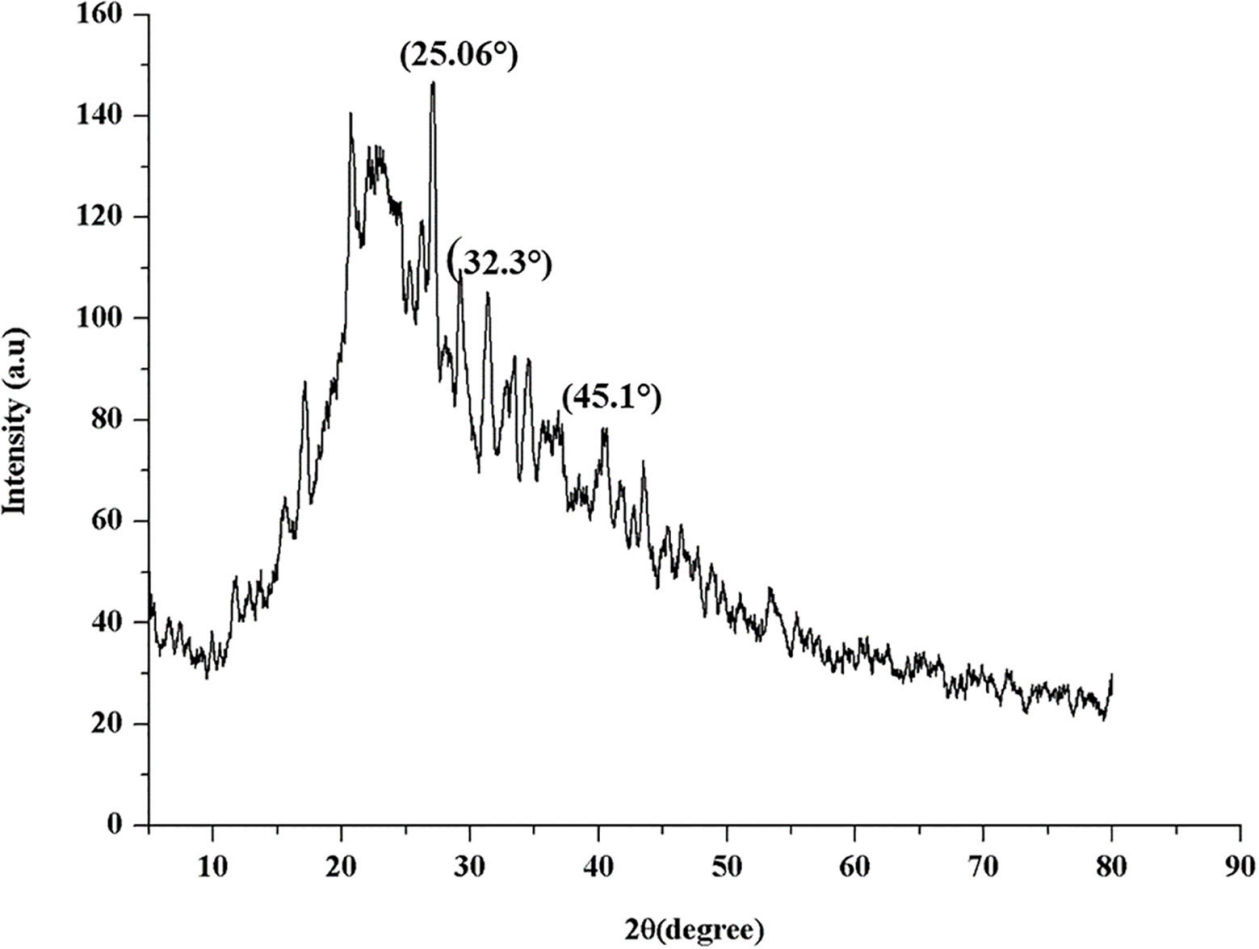
XRD analysis of β-glu-n.

The X-ray diffraction (XRD) analysis of β-glu-n depicted prominent diffraction peaks at 22θ = 25.06°, 32.3°, and 42.1° (**Figure 5**). These peaks are indicative of the crystalline nature of the β-glu-n, aligning with characteristics patterns previously studies for polysaccharides-based nanoparticles. Such crystalline features are commonly observed in β-glucan, as reported previous study (Elnagar et al., 2021).

### 3.2 Assessment of β-glu-n action on tomato wilt (*F. oxysporum*) disease

**Figure 6:**
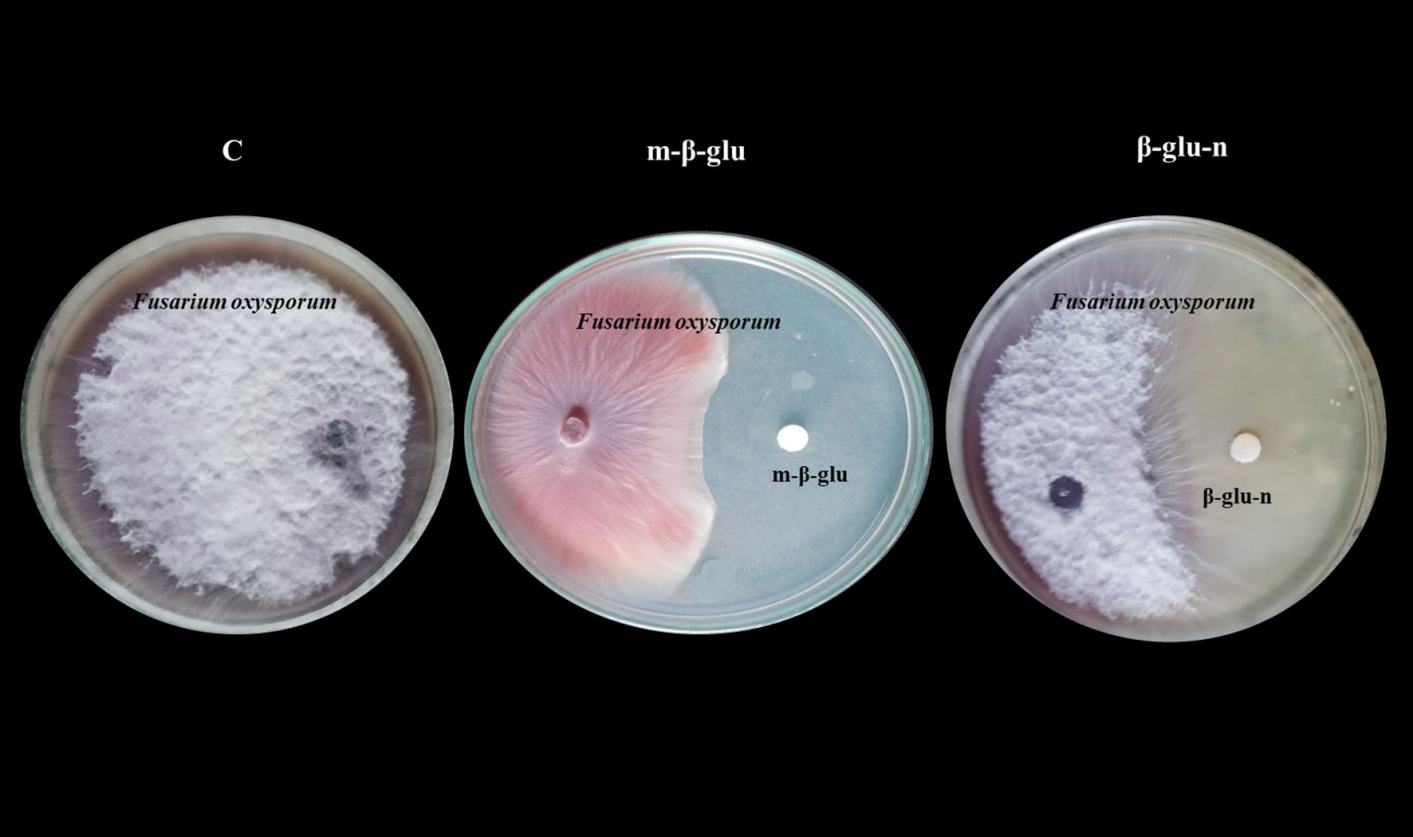
Inhibitory effect of m-β-glucan and β-glu-n on tomato wilt (*F. oxysporum*). C-Control- No Inhibitory effect, m-β-glu-62.35% growth inhibition. β-glu-n-92.67% growth inhibition.

The *in vitro* mycelial growth of *F. oxysporum* significant inhibition when treated with β-glu-n at a concentration of 50 µL. β-glu-n demonstrates a remarkable ability to inhibit the growth of *F. oxysporum* by 92.67%, while the mycelial glucan exhibits an average inhibition rate of 62.35%. No inhibition was noted in the control plates. (**Figure 6**). The results demonstrated that β-glu-n showed enhanced effectiveness against *F. oxysporum* under *in vitro* conditions. The presence of positively charged β-glucan molecules could be a contributing factor, as their interaction with negatively charged membranes appears to be one of the potential mechanisms involved. Previous studies suggested that the compact structure of β-glu-n may facilitate its passage through fungal cell walls, allowing it to attach to DNA and proteins, potentially hindering DNA replication, leading to cellular dysfunction and death. Moreover, the interaction with the cell wall can stimulate the production of reactive oxygen species (ROS) within the fungal cell further contributing to oxidative stress and cellular damage (Kamiński et al., 2022). This result aligns with previous studies indicating that β-glucan nanoparticles exhibit antifungal activity against the phytopathogen *P. aphanidermatum* (Anusuya and Sathiyabama, 2014).

**Figure 7:**
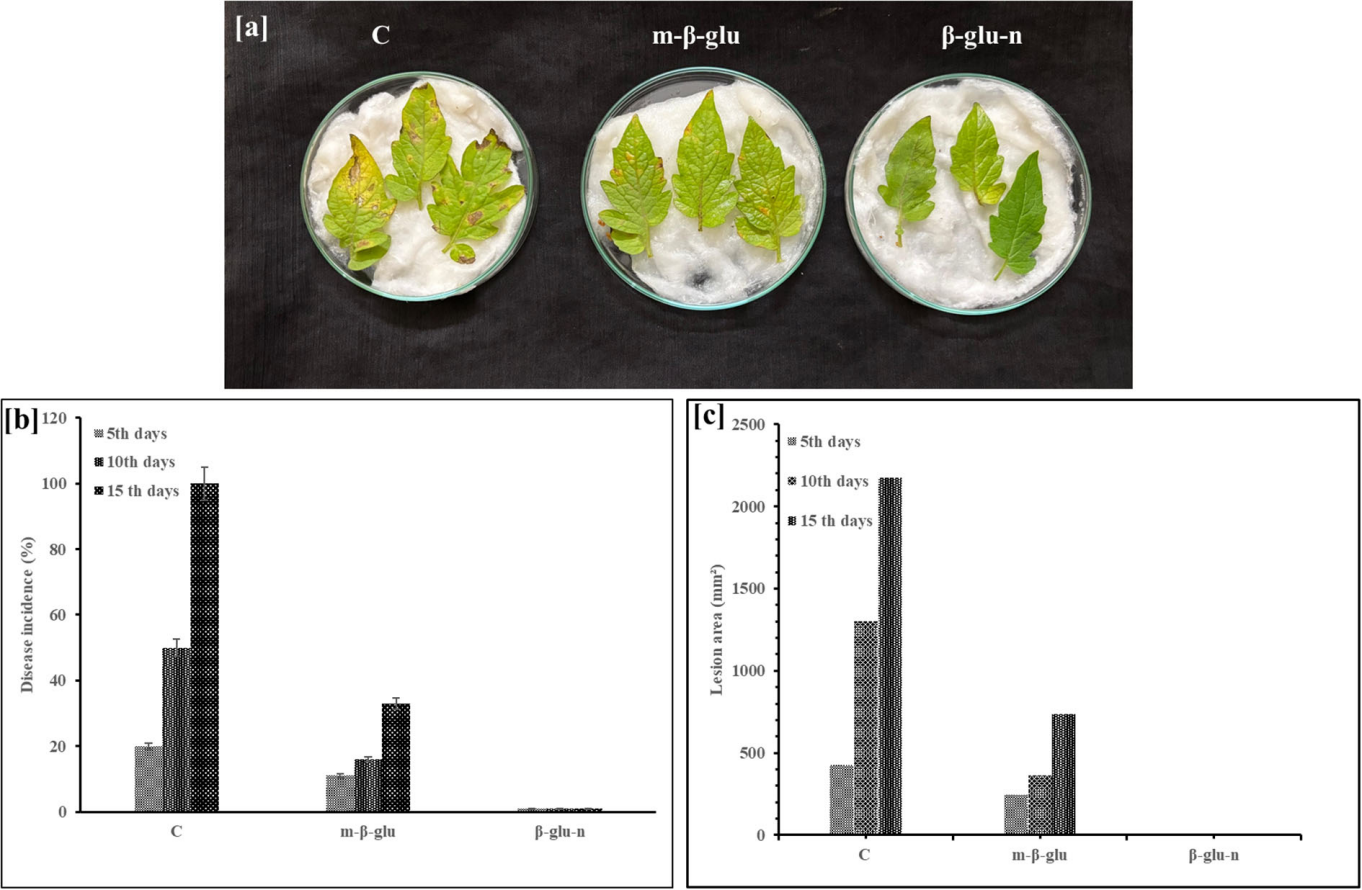
**(a, b)** Suppression of wilt disease on detached leaves of tomato plants. **(a)** C-Control - no suppression of wilt disease; m- β-glucan - 50% suppression of wilt disease; β-glu- n - total suppression of wilt disease. **(b)** Wilt disease incidence percentage on leaves observed on the 5th, 10th, and 15th days for control, m-β-glu, and β-glu- n. **(c)** Symptoms of wilt disease in the lesion area (mm²) 5, 10, and 15th day control, m-β-glu, and β-glucan- n.

The detached leaf techniques provide an *in vivo* environment for the interacting plant pathogens, resulting in effects comparable to those reported in whole plants (Chandra et al., 2015). Symptoms of wilt were observed in leaves treated with m-β-glu after 5 days of infection, with half of the leaf surface exhibiting disease by day 10. In the control group, the symptoms of the disease manifested after a period of two days. Additionally, the presence of the disease (100%) was evident after 15 days in the control group and continued to advance. Leaves that were treated with m-β-glu exhibited a reduction of over 50% in wilt disease in comparison to the untreated infected control. In leaves treated with β-glu-n, a total suppression of wilt disease was noted throughout the entire experimental period (**Figures 7a–c** **).** The results affirm the bioactive properties of β-glu-n. This study represents the first investigation of β-glu-n’s ability to reduce wilt disease in tomato plants. Polysaccharide-based nanocomposites, including fungal cell wall glucan and chitosan, have the ability to infiltrate the plant tissues due to their small size, gaining entry through stomata, wounds, trichomes, and various other points of access (Fernández and Eichert, 2009; Anusuya and Sathiyabama, 2014; Su et al., 2019). Stomata act as an important route for the absorption of nanomaterials (Ali et al., 2011; Ashok Kumar et al., 2014). Tomato plants have stomata on both surfaces (Henningsen et al., 2023), which may have enabled the rapid absorption of β-glu- n. Upon entering, it may trigger the immune response, thereby controlling the growth of invasive plant pathogens alongside with its bioactive properties (Fuertes-Rabanal et al., 2024). Additionally, the increased quantity of superficial functional structures found in β-glu-n enhances its interaction with plant cells.

### 3.3 Assessment of β-glu-n effect on tomato plant growth condition

**Figure 8:**
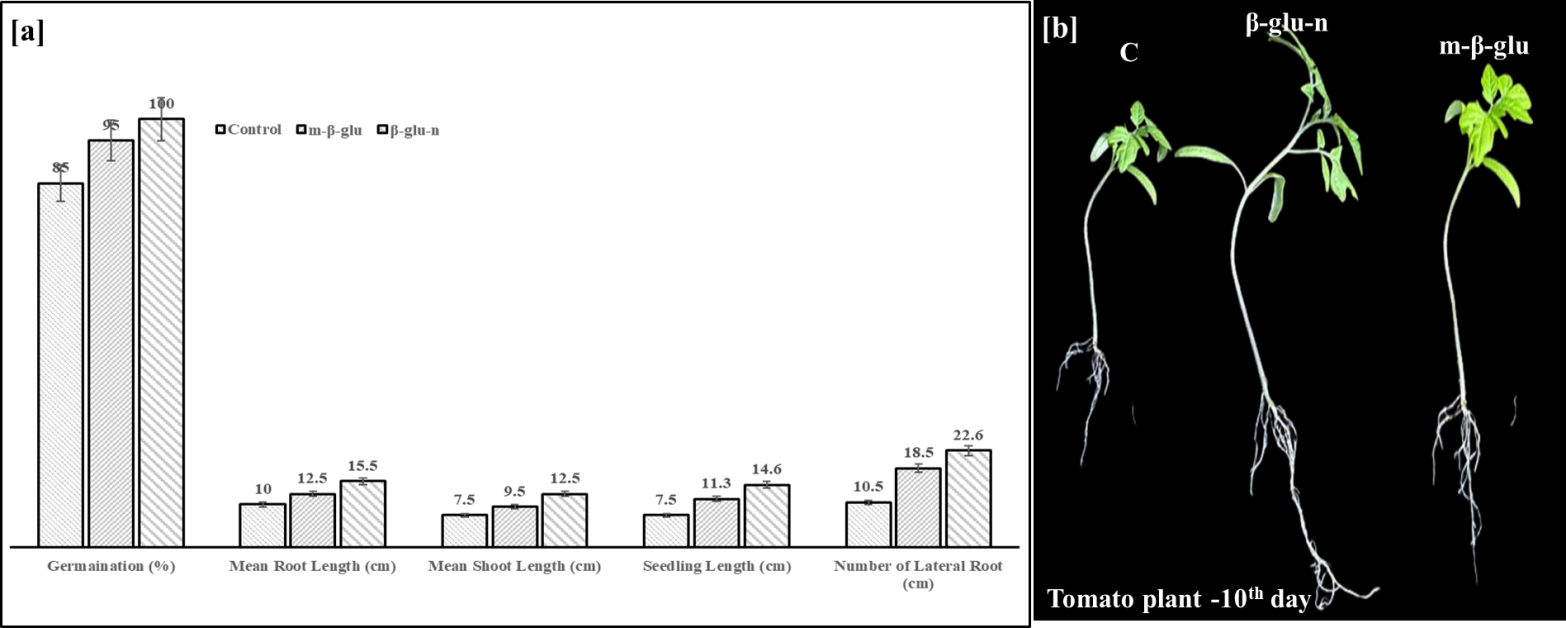
**(a, b)** Effect of β-glu-n on seed germination (%), mean shoot length (cm), mean root length(cm), seedling length and number of lateral roots in tomato plants **(a)**. Effect of β-glu-n and m- β-glu on tomato seedling growth **(b)**.

The tomato seeds treated with β-glu-n and m-β-glu exhibited favourable morphological effects, including improved percentage of germination, increased root and shoot length, a higher seed vigor index, and more effectively vegetative growth of seedlings. Seeds treated with β-glu-n displayed a 100% germination rate and improved seed vigor index (2060), compared to 95% in m-β-glu with seed vigor index (2010) followed by the 85% in control group with a lower vigor index (1302) (**Figure 8a**). **Figure 8b** illustrates that seedlings treated with β-glu-n and m-β-glu exhibited notably longer shoots and roots in comparison to the control group. Additionally, administering β-glu-n led to more lateral root growth in tomato seedlings. The results suggest the β-glu-n treatment demonstrated improved effectiveness in promoting tomato plant growth. Seeds treated with nanoparticles have demonstrated enhanced vigor and improved seedling development across various crops (Arruda et al., 2015). Ultimately, the β-glu-n treatment enhanced tomato plant growth significantly. β-glu-n is absorbed by seeds and transported during photosynthesis. Tan et al. (2012) reported that applying uniconazole-silica nanoparticles to *Oryza sativa* increased the number of roots, root length, and plant fresh weight. Exposure to nanoparticles resulted in increased shoot length as well as a greater growth rate in root tissue, demonstrating an improvement in lateral root growth when compared to the control group. Root lengthening acts as a more precise indicator for assessing the phytotoxic impacts of nanotechnology (EI-Temsah and Joner, 2012). At a root plane, the connections of the rhizoderm is and lateral roots may enable access to nanomaterials, particularly near the root tip, while the upper regions stay impermeable due to the deposition of suberin on the outer layer (Chichiriccò and Poma, 2015). Additionally, the negatively charged nanoparticles are swiftly transported to the bottom of the essential cylinder, enabling their ascent to the plant’s aerial part (Avellan et al., 2017; Zhao et al., 2017).

**Figure 9:**
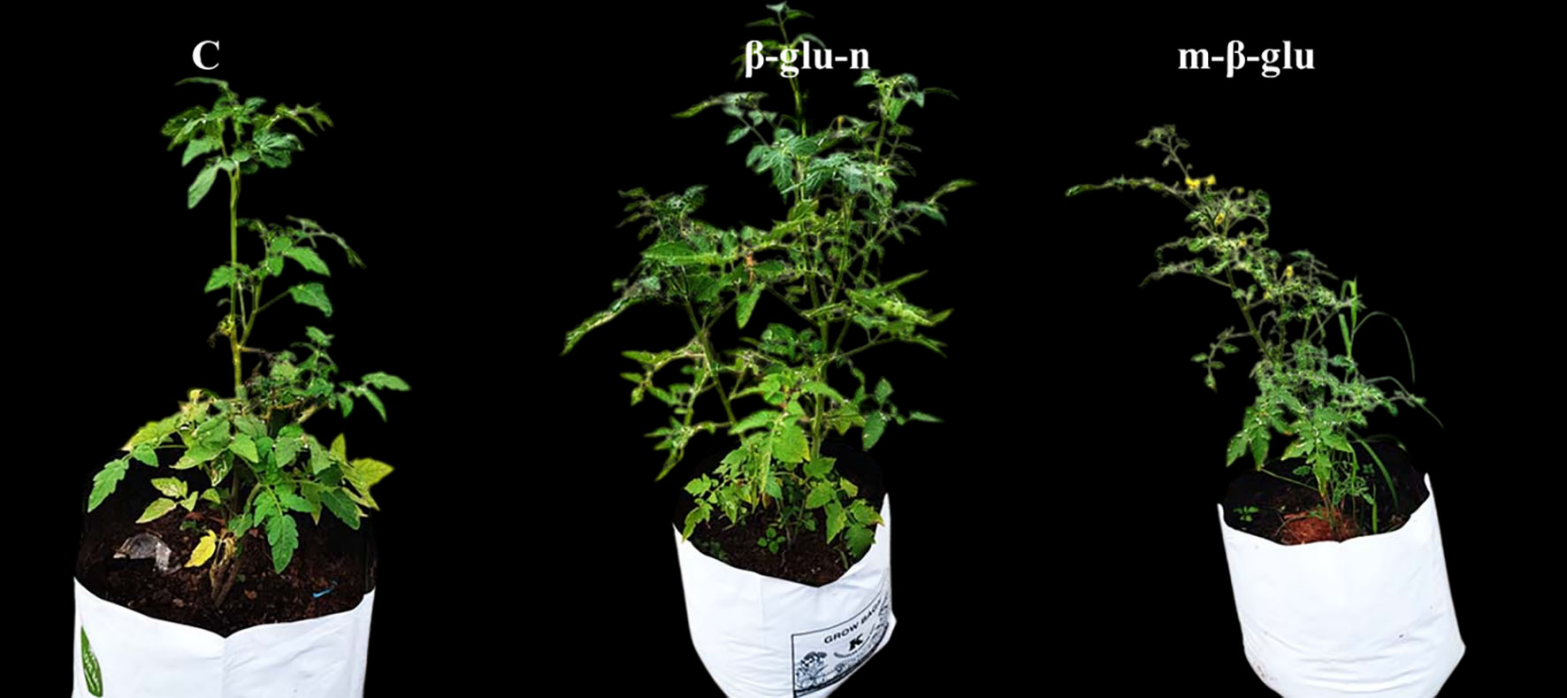
Effect of β-glu-n and m- β-glu on tomato plant growth development under glass house condition-control, β-glu-n and m- β-glu.

In evaluating the growth of tomato plants, those treated with β-glu-n and m-β-glu exhibited enhanced growth in plant growing bags, showing no adverse effects, whereas control plants exhbits the leaf lesion symptoms with decreased growth (**Figure 9**). Chemical fungicides, along with metal or carbon-based nanoparticles, were utilized in the soil as fertilizers for agricultural plants (Gao et al., 2006; Verma et al., 2018). Considering the significant evidence, the use of nanoparticles and chemical fungicides as soil additives may present a threat to soil microbes, consequently limiting the application of metallic nanoparticles in agriculture practices (Simonin et al., 2016; Goncalves et al., 2017). Biopolymer/natural nanocomposites are regarded as significantly less harmful because of the slow-release phenomena, and their enduring impact has been evidenced in agricultural crop plants. The use of glucan-based nanocomposites in agricultural crops improves the length of leaves, shoots, and roots (Anusuya and Sathiyabama, 2014). The treatment with glucan nanocomposite may have increased levels of gibberellic acid, which play a key role in shoot development (Stepanova et al., 2007). Glucan nanoparticles administration likely interacts with ethylene inhibition, leading to a reduction in abscission occurrence. This interaction significantly contributes to the observed increase in leaf number (Seif et al., 2011).

### 3.4 Examination of the protein profile in β-glu-n-treated tomato plants’ growth

**Figure 10:**
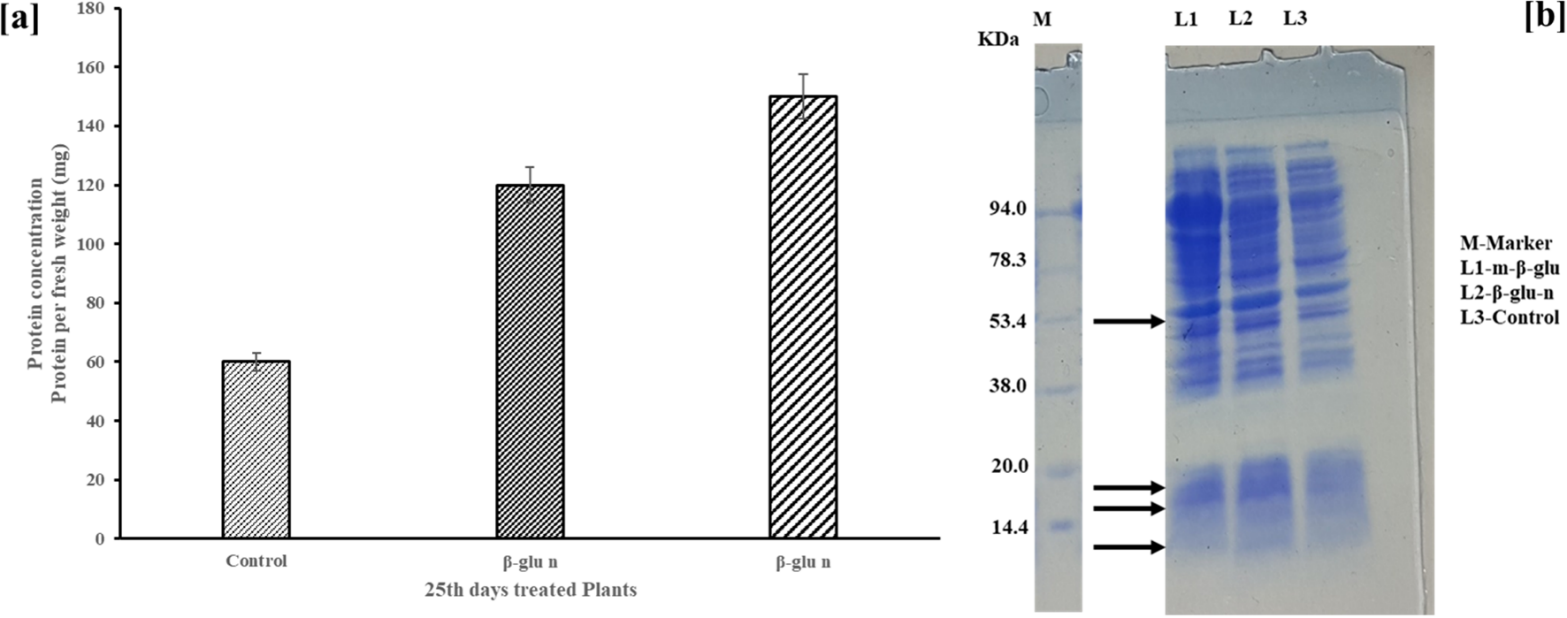
**(a, b)** Protein levels of control, and treated (β-glu-n, m- β-glu-n) tomato plant leaves **(a)**, The SDS-PAGE analysis of the protein in the tomato leaves on the 25th day displayed M-Marker- standard protein marker, L1- β-glu-n, L2- m- β-glu, L3-Control **(b)**.

The leaves of the treated (β-glu-n and m-β-glu) crops exhibited an increase protein content when compared to the control (**Figure 10a**). Electrophoresis was utilized to assess ribulose bisphosphate carboxylase (Rubisco), a crucial enzyme in the process of photosynthesis (Aranjuelo et al., 2005). The SDS-PAGE analysis of the protein in the tomato leaves on the 25^th^ day showed that both the control and treated tomato plants displayed polypeptides ranging from 25 to 90 kDa comparison with standard protein marker. Furthermore, the β-glu-n treated tomato plants leave exhibited the presence of three novel polypeptides with molecular mass of 14, 19, and 23 kDa. The treated (β-glu-n and m-β-glu) seedlings exhibited elevated levels of a molecular mass of 54.5 kDa when compared to the control group (without treatment). (**Figure 10b**). The structure comprises two major subunits, each with a weight ranging from 50 to 55 kDa, in addition to two smaller subunits that range from 12 to 18 kDa. The combined components could account for roughly half of the total soluble protein and a quarter of the total nitrogen present in leaf tissue (Tabita et al., 2007). The function of Rubisco is to boost the overall photosynthetic efficiency of cereal crops in various climates (Parthasarathy et al., 2023). Nitrogen-courting rice and Rubisco have demonstrated their ability to improve the crop development and leaf photosynthesis (Makino, 2003; Perdomo et al., 2017). Nanoparticles (NPs) influence Rubisco dynamics in dicot plants by delaying its natural degradation post -leaf maturation, thereby sustaining photosynthetic activity beyond typical growth phase. This effect is confirmed through electrophoretic analysis showing persistent Rubisco presence even 25 days after Nps exposures.

This research indicates that β-glu-n can be synthesized using TPP, demonstrating its potential applications in agriculture for enhancing plant growth and managing diseases.

Further studies are necessary to thoroughly assess the toxicity and safety considerations, molecular mechanism of biosynthesised β-glu-n before its widespread application in the agricultural sector. In addition, understanding the environmental fate and potential accumulation of β-glu-n in the ecosystem is crucial for developing safe and effective agricultural uses. Consequently, through safety assessment and regulatory evaluations are essential steps prior to endorsing the large-scale use of biosynthesised β-glu-n in agricultural field.

## 4 Conclusion

To sum up, the investigation outlines the synthesis of β-glucan nanoparticles using TPP. The β-glucan extract is derived from the marine algal associate fungi (*A. awamori*) and confirmed via ^1^H NMR. The structure of the β-glu-n was analyzed using ^1^H NMR and IR spectrum. The glucan nanoparticles that were synthesized underwent characterization via HR-TEM, DLS, and XRD analyses. The zeta potential of the biosynthesized β-glu-n was measured at -38.40 mV, suggesting its colloidal stability in a water solution. β-glu-n demonstrated inhibitory activity against the highly destructive tomato wilt pathogen *F. oxysporum* under *in vitro* conditions. The detached leaf method demonstrated suppression in wilt diseases in tomato leaves and indicates that β-glu-n may be effective in managing tomato pathogens, enhancing plant growth and protein content. Thus, this study demonstrates that β-glu-n, when used in crops seeds and fertilizer amendments, can enhance productivity in modern agriculture and serve as an innovative solution for tomato wilt disease. Additional research into the biochemical and molecular factors in field conditions is essential to confirm the precise mechanisms.

### 4.1 Statistical analysis

The data was analyzed using one-way ANOVA to determine the significance of individual differences at p < 0.01 and 0.05 levels. All statistical analyses were carried out using SPSS 21 software support version 5.0.

## Data Availability

The datasets presented in this study can be found in online repositories. The names of the repository/repositories and accession number(s) can be found in the article/**Supplementary Material**.
