# Supplementary material for: Aspergillus-derived β-glucan nanoparticles: a dual strategy for Fusarium Wilt management and tomato plant growth enhancement
Source: Front Plant Sci. 2025 Jul 30;16:1611582. doi: 10.3389/fpls.2025.1611582 (PMC12343568; doi:10.3389/fpls.2025.1611582)

**Supplementary Figure S1.** *A. awamori*, a marine algal-associated fungal, was isolated with black colonies in PDA media and observed under a 100× microscope.


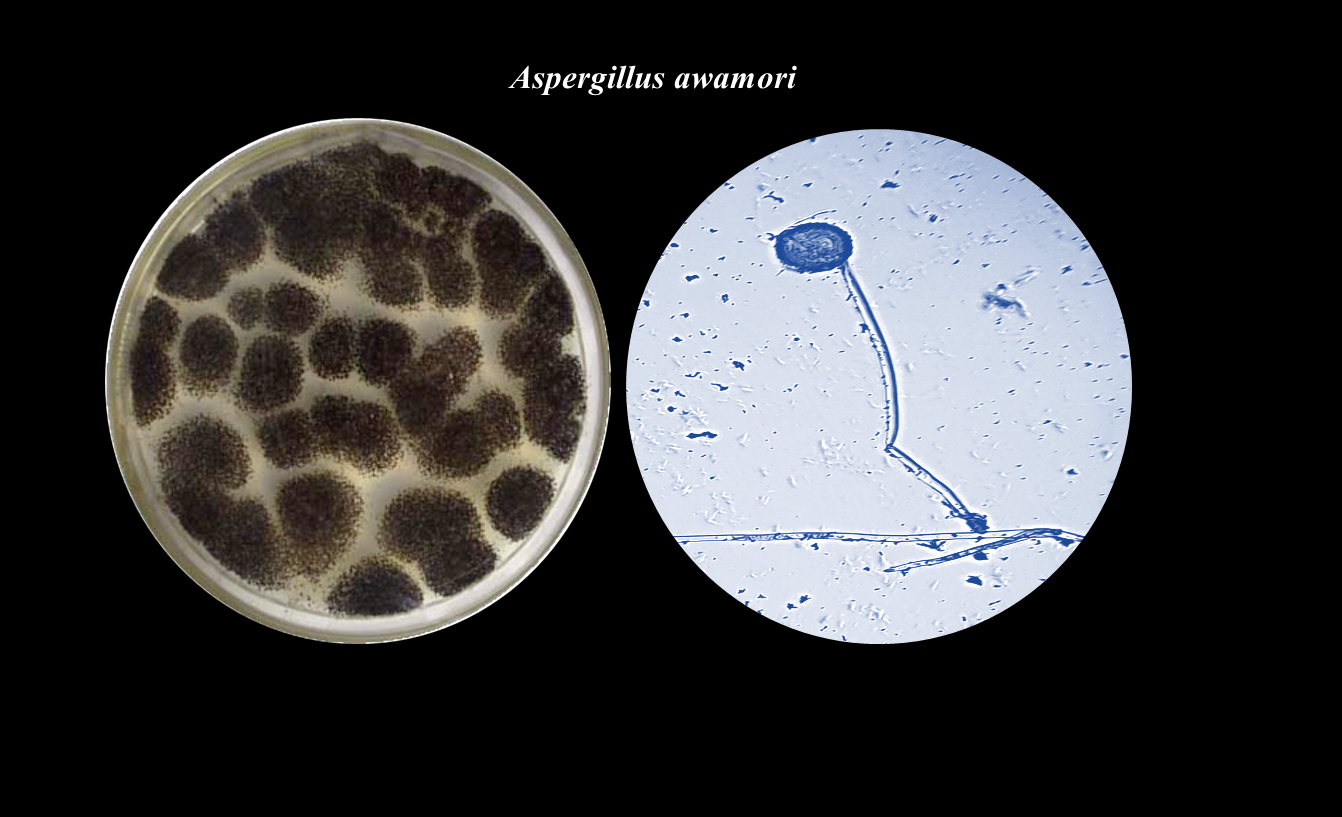


**Supplementary Figure S2.** Phylogenetic tree construction of the ITS gene sequences of *A. awamori*. *Denotes the fungus under investigation.

**
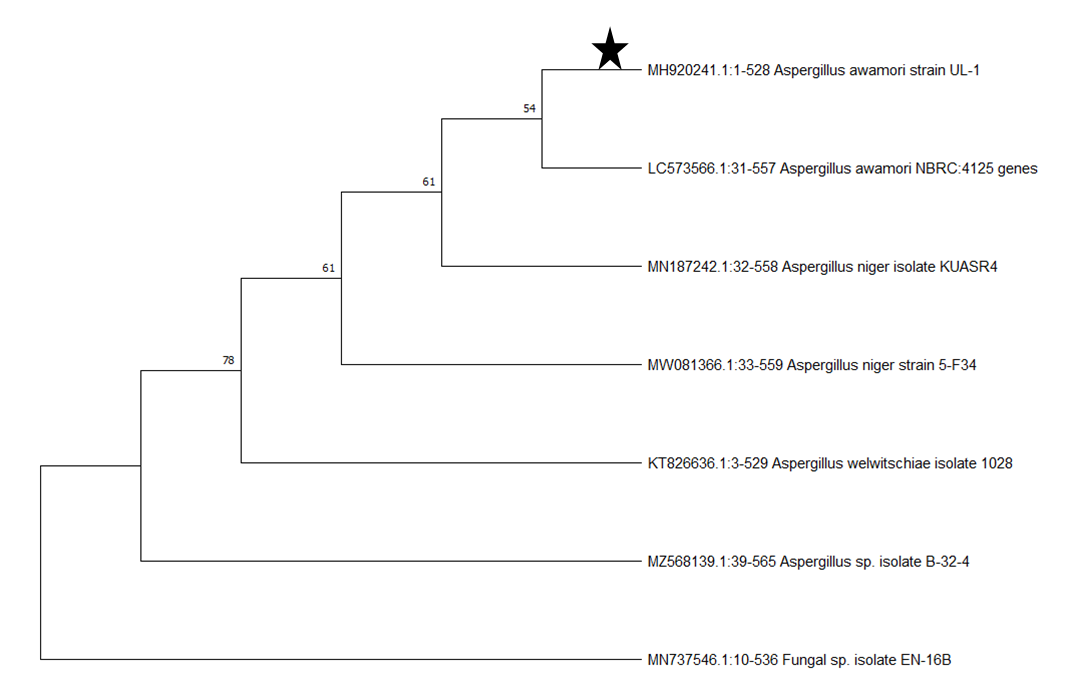
**

**Supplementary Figure S3.** UV spectrum analysis of β-glu-n (389.45nm).


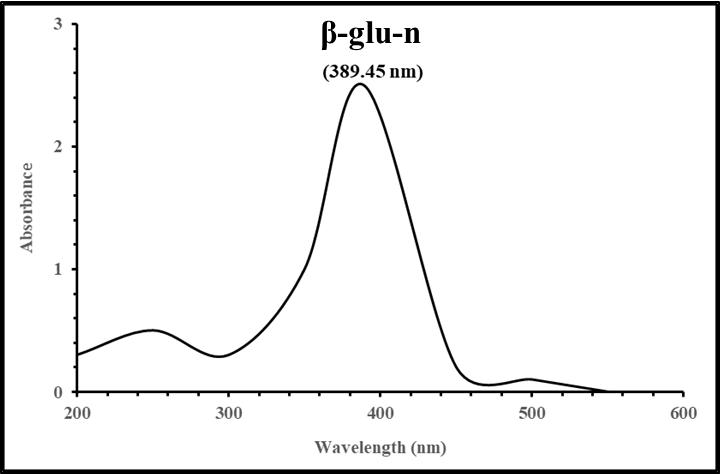

Supplement: Supplementary file 1 [file DataSheet1.docx]
